# Anthracological study of a Chalcolithic funerary deposit from Perdigões (Alentejo, Portugal): A new analytical methodology to establish the wood burning temperature

**DOI:** 10.1371/journal.pone.0287531

**Published:** 2023-07-07

**Authors:** Ginevra Coradeschi, Nicasio T. Jiménez Morillo, Cristina Barrocas Dias, Massimo Beltrame, Anabela D. F. Belo, Arturo J. P. Granged, Laura Sadori, António Valera

**Affiliations:** 1 HERCULES Laboratory, University of Évora, Évora, Portugal; 2 CityUMacau Chair in Sustainable Heritage, Évora University, Évora, Portugal; 3 MED – Mediterranean Institute for Agriculture, Environment and Development & Department of Biology, School of Science and Technology, Mitra (UE Campus), Évora, Portugal; 4 Institute of Natural Resources and Agrobiology of Seville (IRNAS-CSIC), Seville, Spain; 5 Chemistry and Biochemistry Department, School of Sciences and Technology, University of Évora, Évora, Portugal; 6 Department of Applied Physics I, University of Sevilla, Seville, Spain; 7 Med_Soil Research Group, Faculty of Chemistry University of Sevilla, Seville, Spain; 8 Department of Environmental Biology, Sapienza University of Rome, Rome, Italy; 9 Archaeological Research Unit of Era Arqueologia S.A., Lisboa, Portugal; 10 ICArEHB Algarve University, Gambelas Campus, Algarve University, Algarve, Portugal; New York State Museum, UNITED STATES

## Abstract

Anthracological analyses of charcoal samples retrieved from Pit 16 of Perdigões (Reguengos de Monsaraz, Portugal), a secondary deposition of cremated human remains dated back to the middle of the 3^rd^ millennium BC, enabled the identification of 7 different *taxa*: *Olea europaea*, *Quercus* spp. (evergreen), *Pinus pinaster*, *Fraxinus* cf. *angustifolia*, *Arbutus unedo*, *Cistus* sp. and Fabaceae. All *taxa* are characteristic of both deciduous and evergreen Mediterranean vegetation, and this data might indicate that the gathering of woods employed for the human cremation/s occurred either on site, or in its vicinity. However, considering both the large distribution of the identified *taxa* and data about human mobility, it is not possible to conclusively determine the origin of the wood used in the cremation(s). Chemometric analysis were carried out to estimate the absolute burning temperature of woods employed for the human cremation/s. An in-lab charcoal reference collection was created by burning sound wood samples of the three main *taxa* identified from Pit 16, *Olea europaea* var. *sylvestris*, *Quercus suber* (evergreen type) and *Pinus pinaster*, at temperatures between 350 and 600 °C. The archaeological charcoal samples and the charcoal reference collection were chemically characterized by using mid-infrared (MIR) spectroscopy in the 1800–400 cm^-1^ range, and Partial Least Squares (PLS) regression method was used to build calibration models to predict the absolute combustion temperature of the archaeological woods. Results showed successful PLS forecasting of burn temperature for each *taxon* (significant (*P* <0.05) cross validation coefficients). The anthracological and chemometric analysis evidenced differences between the *taxa* coming from the two stratigraphic units within the Pit, SUs 72 and 74, suggesting that they may come from two different pyres or two different depositional moments.

## 1. Introduction and research aims

**Fig 1:**
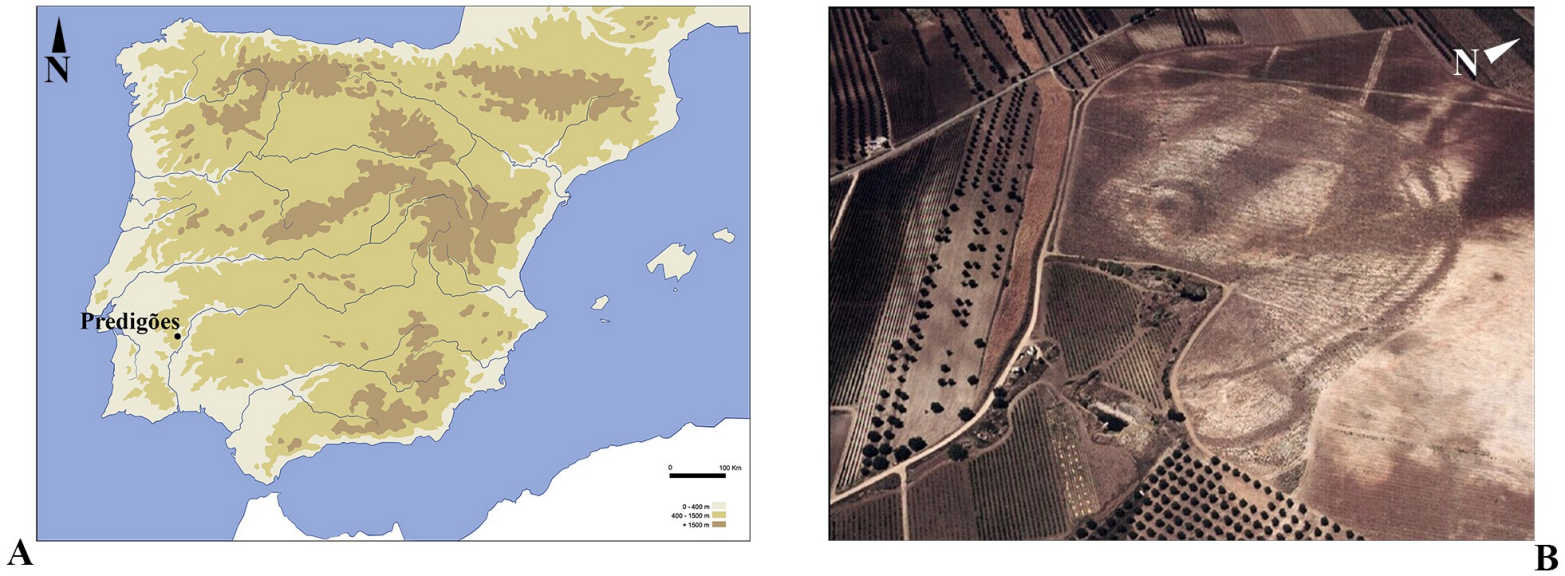
A. Location map of Perdigões archaeological site in the Iberian Peninsula, B) Aerial photo of Perdigões ditch enclosures.

Perdigões is a large set of ditched enclosures, nowadays classified as a National Monument, located in Reguengos de Monsaraz municipality, Évora district, in the inner Alentejo region, centre-southern Portugal (Fig 1A and 1B). It holds at least 16 ditches and a megalithic cromlech, occupying an area of ca. 18 ha. The archaeological exploration of the area started back in 1997 and the site had a long and complex diachrony, extending from 3400 BCE (Middle—Late Neolithic transition) to 2000 BCE (Early Bronze Age).

**Fig 2:**
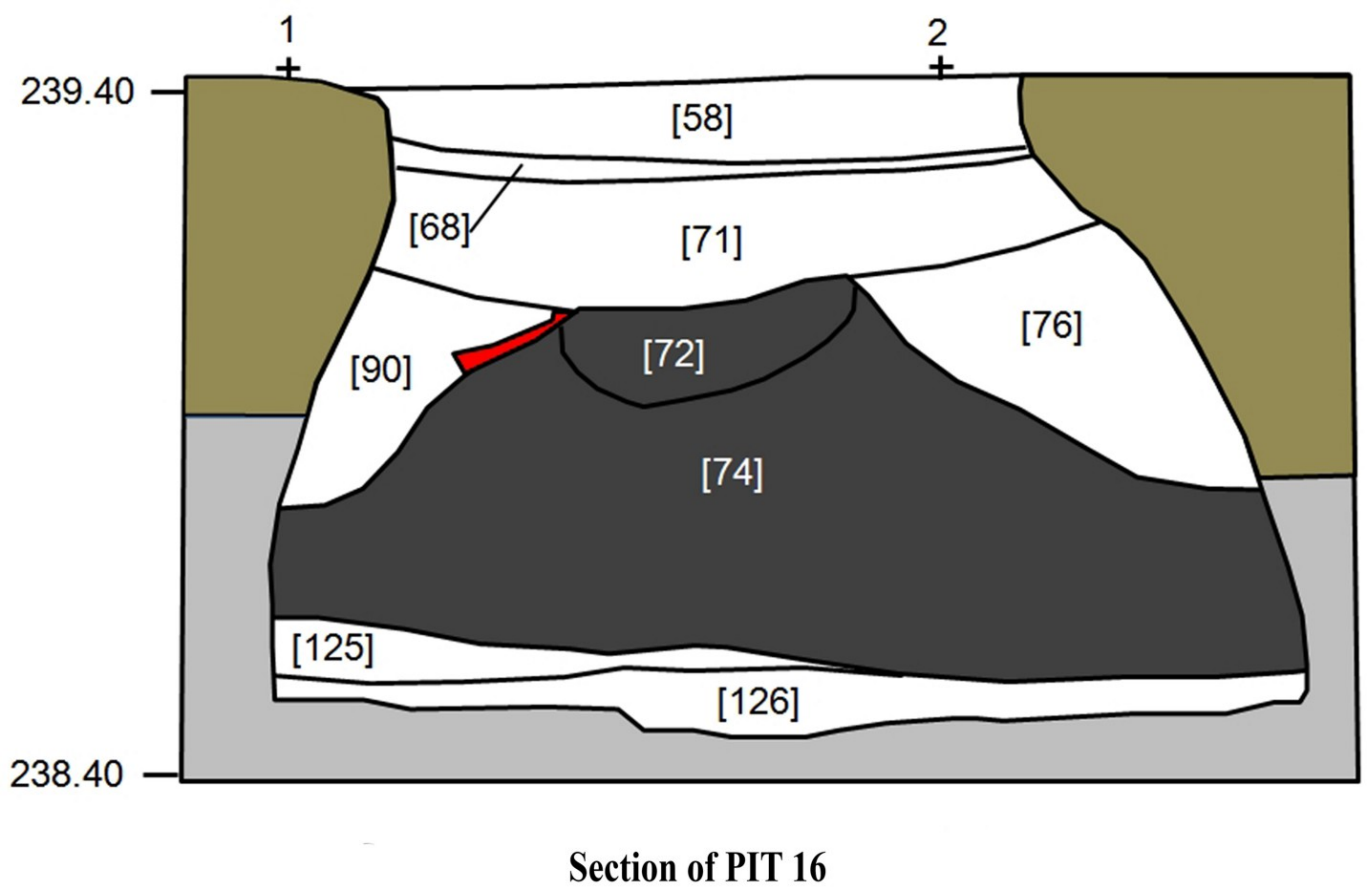
Section of Pit 16. In dark grey: the two stratigraphic units (SUs 72 and 74) containing human remains associated with charcoals and the grave goods.

Interpreted as a ceremonial and aggregation centre, these ditched enclosures have several funerary structures, revealing diversified funerary practices in terms of architecture, location within the site, body treatment and votive materials [1–3]. The archaeological material under study was recovered in Pit 16 (middle 3^rd^ BC), located in the central area of the enclosures [2, 3]. Several stratigraphic units (SU) were identified during the archaeological excavation, and those that contain human remains were designated SU 72 and 74 (Fig 2).

In southern Portugal and more generally throughout the Iberian Peninsula, there are no other Chalcolithic funerary contexts with the characteristics of Pit 16. Burial rituals in this area during this period were quite heterogenous, including dolmens, tholoi, pits, ditches and hypogea [4, 5]. Cremation seems to have been used only for a few individuals per site and wasn’t the most common practice [6]. The different ways of human bodies processing, and the type of funerary structures and grave goods differ between cremation and non-cremation funerary contexts. Some authors [7] argued that these differences may have been related to different social and cultural backgrounds and to social emulation processes.

Perdigões, along with other recent archaeological discoveries, led to a revision of the Megalithic literature [1, 2, 4–11] and, at present, a more dynamic picture of the Iberian Chalcolithic funerary world is available. The reinterpretation of the world of death within this particular chronology has fostered the production of a number of studies on anthropological and archaeozoological remains recovered in these contexts, and the cultural materials associated with them [1, 4, 12–23].

Overall there is a general lack of studies of funerary charcoals across Europe, with only a few studies published about Prehistoric sites [24–27] with examples for Great Britain and Ireland [28–30]; starting from the Roman Period, studies become more frequent and systematically conducted [31–39].

In the literature a charcoal assemblage deriving from human cremation deposits is considered a single or a short time span episode [30]. The main goal of the study of these types of archaeological charcoals is to get paleo-ethnographic information, wherein the relationship between humans and their surrounding nature is investigated [25]. The analysis of charcoals from these types of funerary contexts may reflect rational and less rational human choices, linked with a ritual character of the site, where the taxonomic selection of woods employed for the pyre may have a cultural meaning [25, 30, 34, 35, 40, 41]. Indirectly, the interpretation of human cremation contexts is tied to the study and to the interpretation of the charcoal assemblage. On the other hand, as some authors have already mentioned [30], charcoal is the remainder of the funeral pyre, being the most direct way to get a glimpse on the ritual of body cremation.

The aims of this study are:

- To characterize the different wood *taxa* from Pit 16.
- To evaluate the absolute burn temperature of the tree main wood *taxa* identified from the Pit.
- To obtain information regarding this unusual Chalcolithic funerary context, exploring the existence of one or different moments of secondary deposition of human bodies.

Two different approaches have been used to achieve these goals. The first one includes the anthracological analysis of the charcoal assemblage, and the second one comprises the FT-MIR/chemometric analyses of archaeological charcoals and sound wood samples (of the same species identified) combusted at different temperature (i.e., formed under controlled temperature and atmosphere using a muffle furnace in the laboratory).

We consider this approach a good option to successfully evaluate the charcoal assemblage from Pit 16. Results were useful for a better understanding of the archaeological context and the related ritual/s.

### 1.1. Checking out different techniques for the study of the archaeological charcoal absolute burning temperature

The partial combustion process of wood either in a forest fire, in a bonfire or in a funerary pyre is well known. It normally produces charred material which contains a significant amount of refractory carbon (C) [42, 43]. This is commonly known as “black carbon” or charcoal [44]. This material is defined as a continuum of thermally altered products, ranging from slightly charred still degradable biomass, to highly condensed, refractory soot [45, 46]. The charcoal particles are chemically heterogeneous, but typically they are made up of a conspicuous amount of unspecific chemical aromatic structures [46].

The concentration of aromatic compounds within the chemical structure of the charcoal particles is significantly associated with direct and indirect factors. Direct factors are linked to combustion characteristics, including temperature, fire permanence and heating rate (°C/min). On the other hand, indirect factors are connected to the fuel (wood) properties, like the type of wood, size, wood resilience and moisture [47–49]. Thus, chemical changes in the continuum charcoal structure can be identified, and are influenced by the combustion temperature and the molecular composition of the pyrogenic material [50–52].

Currently, different techniques are available for the charred organic matter evaluation of complex matrices such as soils and sediments, as well as to assess their burning temperatures. One of these techniques is the reflectance, which is considered a valuable tool to estimate the burn temperatures of modern and archaeological charcoal using a Reflected Light Microscope [49, 53]. Another analytical technique is the Fourier Transform Mid-Infrared (FT-MIR) spectroscopy, which is micro-destructive and employed for the chemical characterization of complex matrices in archaeological samples, like wood charcoal particles and bone collagen [54–56].

The combination of FT-MIR spectroscopy and multivariate analytical techniques, such as Partial Least Squared (PLS) regression [57], has demonstrated to be a powerful tool for the evaluation and prediction of combustion temperature of organic matter and the composition of pyrogenic carbon in soils [44, 58]. Recently, some researchers have studied burned wood samples in terms of the Variable Importance Projection (VIP), which has allowed them to assess MIR bands significantly linked with dependent variables [44, 58].

## 2. Materials

### 2.1. Archaeological context of the charcoal samples

Pit 16 is interpreted as a secondary funerary collective deposition, a singular funerary context amongst the Iberian Chalcolithic. The pit contained human remains of at least 9 individuals (6 adults and 3 sub-adults), mixed with other burnt artefacts (faunal remains, fragments of pottery, ivory idols, and arrowheads) as well as a large number of charcoals. The pit is circular, with a profile shrinking at the top, presenting a diameter of 0.92 m at the top and 1.50 m at the base, being 0.90 m deep (Fig 2). In total, nine different stratigraphic units (SU) were identified, corresponding to three phases of deposition. The first phase corresponded to the initial filling of the pit, where two thin layers (SU126 and SU125) were formed by some residual materials, namely pottery sherds. The second phase corresponded to the secondary deposition of the cremated remains. A conic shaped deposit was formed, indicating the dumping of the remains into the pit (probably from containers). This deposition was subdivided into the two aforementioned stratigraphic units, SU72 and SU74, the later one being less compact and dustier. Both sediments were dark grey with a lot of charcoal and ashes, involving an abundant amount of human remains (5000 fragments), some cremated faunal remains and burned archaeological materials (pottery sherds, ivory schematic figurines, a copper awl, stone arrowheads) [2, 3, 59–61]. Nevertheless, whether the two stratigraphic units represent a single deposition moment or not, is not clear. The third phase corresponds to the final filling of the pit, with the depositions of several layers with no human remains, incorporating mainly faunal bones and pottery sherds, most of them burned (SU76, SU71, SU90, SU68, SU58).

### 2.2. Current vegetation of the area

Nowadays, in natural areas, the woody vegetation cover is made up mostly of evergreen oaks (*Quercus suber*, *Quercus rotundifolia*, *Quercus coccifera*), *Arbutus unedo*, some *Pinus* spp. and *Olea europaea* var. *sylvestris*, along with many sclerophyll shrubs, like *Cistus* spp., *Rosmarinus officinalis*, *Lavandula* spp., *Myrtus communis*, *Pistacia lentiscus* or *Phillyrea* sp.; some deciduous trees are also present near streams and other watercourses, like *Fraxinus angustifolia*, *Populus* sp., *Salix* sp..

## 3. Methods

### 3.1. Sampling

Pit 16 sampling was performed based on the excavation pursued, which divided the pit into several stratigraphic units. For this work, sediments from the SU72 and the SU74 (Fig 2), corresponding to the secondary deposition of cremated human remains, were entirely sampled.

In the field, archaeologists dry sieved the sediments from SU72 and SU74 using a 2 mm mesh size to isolate human remains [60]. After the bone recovery, sediments were preserved for the anthracological study.

### 3.2. Anthracological analysis

Sediments from SU72 and SU74 (which had been previously dry sieved in the field to recover human remains) were additionally treated and observed in the laboratory using a stereo-zoom microscope (model LEICA M205C equipped with a camera) to remove sand grains, small ceramic, and animal bone remains and to isolate charcoal fragments (i.e., larger than 2 mm) to proceed with the anthracological analysis. A total of 754 charcoals were recovered.

The charcoal fragments were manually fractured to obtain the three anatomical sections (transversal, tangential and radial) under a stereo-zoom microscope (LEICA M205C equipped with a camera). The charcoal’s anatomical features were determined using a reflected light optical microscope (LEICA DM2500 equipped with a camera) at different magnifications (50–1000x). A selection of charcoal fragments was analysed with a Scanning Electron Microscope (SEM) to obtain high-resolution images. Wood atlases [62–65] were used as comparative tools for the charcoal identification, together with an in-lab reference collection of wood specimens. Some other types of observation were also made. When present, fractures in radial directions (radial cracks) [66–71], vitrification, consisting in a variable fusion of some anatomical parts of the wood (which make the wood structure homogeneous) [69–74], and radial groves in the inner part of tracheids cells (known as reaction wood) [69] were recorded.

With respect to the evergreen oak’s wood samples identification, it is significant to reiterate that it was not possible to make any distinction between the evergreen oak tree species. In Portugal, nowadays, as well as in the Mediterranean region, this group includes holm oak (*Quercus ilex* L.), kermes oak (*Quercus coccifera* L.) and cork oak (*Quercus suber* L.) [64, 75].

Regarding the identification of the olive wood samples, the cultivated species (*Olea europaea* var. *europaea*) is not differentiable, at the anatomical level, from the wild one (*Olea europaea* var. *sylvestris*). However, studies concerning the beginning of the cultivation of this plant in the south of the Iberian Peninsula suggest that it started only in the Roman period [76]. Considering the investigation period, charcoal fragments of *Olea* from Pit 16 could probably be ascribed to the wild variety, *O*. *europaea* var. *sylvestris*.

Concerning the identification of the maritime pine wood, archaeobotanical remains showed the presence of this species since the Holocene in the northern Iberian Peninsula, in the mountainous regions of eastern Iberia as well as in the southwestern part of the peninsula [77–79]. This proves the naturalness of this species in these areas [77, 80].

In this work, the identification of charcoals belonging *Pinus pinaster* was based on the presence and distribution of teeth in the radial tracheids, taking into consideration three levels of degree of dentation [64, 81–83].

The identification of Fabaceae wood is considered particularly difficult due the large intraspecific structural variability present in this group [84–86]. Few species of this group were anatomically described [65, 84]. In the present study charcoal samples belonging to this *taxon* were identified only at the family level.

### 3.3. Data quantification

For data interpretation, *taxa* values are interpreted using the relative frequency (%) and the mean values (%U) obtained using the “ubiquity correction” method [87]. The mean value (%U) of a specific *taxon* (A, for example) on *n* different stratigraphic units (SU) is calculated as: %U = %A _(SU1)_ + %A _(SU2)_ + %A _(SUn)_ / *n*. This method performs a correction for ubiquity of the relative frequencies of each taxon. Charcoal size classes (0.3–0.99 cm, 1–1.99 am, and 2–2.99 cm) for each wood *taxon* identified were also collected.

### 3.4. Estimation of the absolute burn temperature of archaeological charcoals

#### 3.4.1. Experimental combustion tests on modern reference material

**Fig 3:**
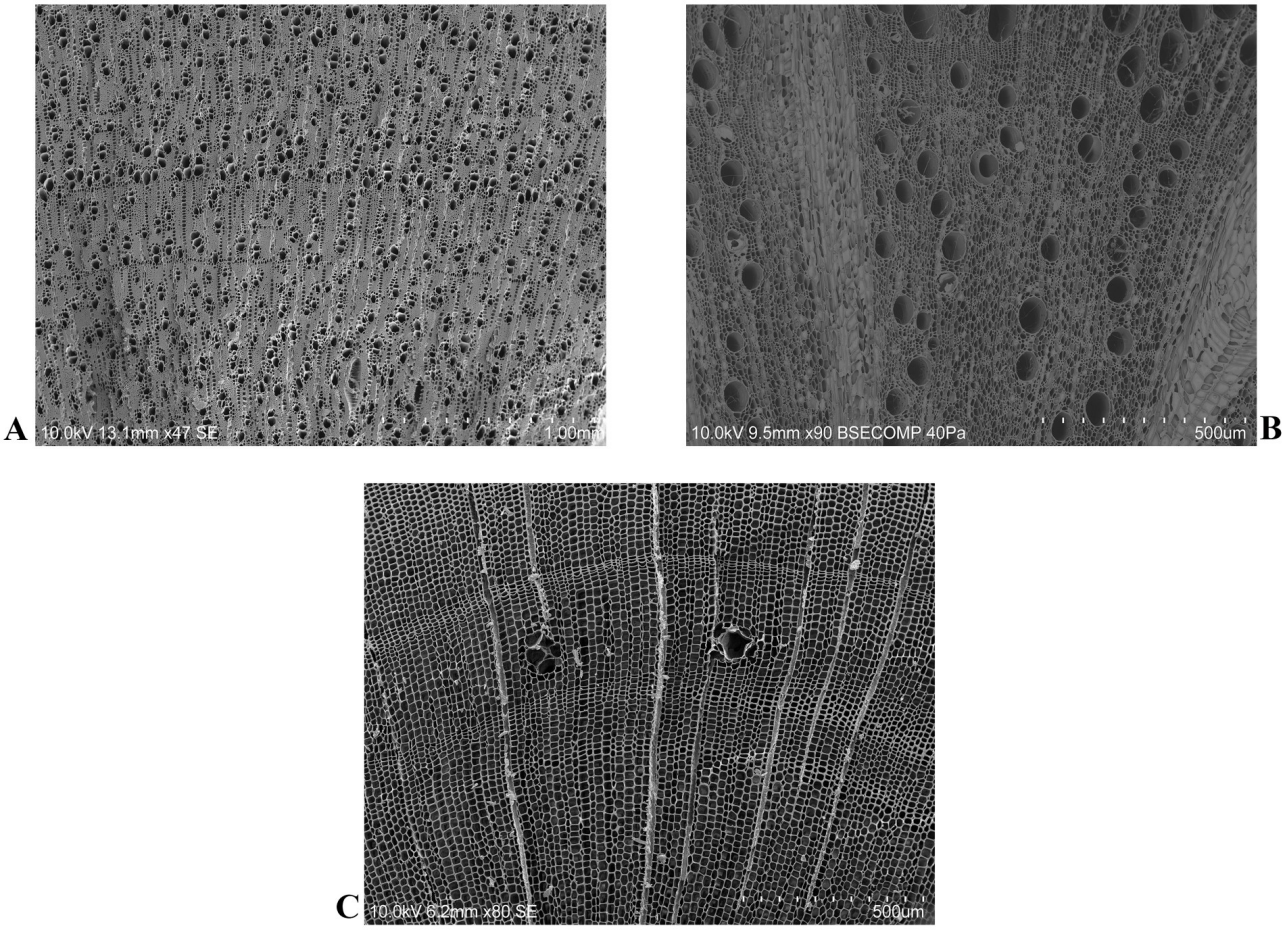
Sound woods transversal anatomical section of *Olea europaea* var. *sylvestris* (A), *Quercus suber* (B), and *Pinus pinaster* (C) heated at 350°C. Samples were photographed with SEM.

To create a statistical model able to estimate the absolute combustion temperature of archaeological charcoals an in-lab charcoal reference collection was created, and different experimental combustion tests were carried out. Sound wood samples of the three main *taxa* identified from Pit 16 with the anthracological analysis, namely olive, evergreen oak, and maritime pine woods have been sampled in the proximity of the archaeological site (Fig 3).

Regarding the olive and considering the investigation period (as already reported), wooden samples belonging to the wild variety, *Olea europaea* var. *sylvestris* has been chosen as reference material.

For the evergreen oaks, samples belonging to *Quercus suber* have been chosen as reference material, considering the abundance of this species in the Alentejo region [88]. After sampling, sound wood samples were cut (dimension: 1 cm^3^, ≈ 3 g) using an automatic saw, then washed with distilled water and dried for 24 h. Samples were then positioned into ceramic crucibles, heated in the absence of oxygen, between 350 and 600°C, the typical burning temperature of wood in natural fires [89]. In total, 6 different heating steps were used, increasing the muffle furnace temperature of 50°C each time.

Samples remained within furnace during 90 min at maximum temperature. For each heating step, two replicates of each taxon were burnt. At the end of the heating period, a sample of each laboratory-based combusted charcoal was photographed by SEM after the gold coating procedure.

#### 3.4.2. FT-MIR spectroscopy analysis of modern and archaeological charcoals

An aliquot from the two charred replicas of each modern wood sample was selected for Fourier Transform Mid-Infrared analysis. The two charred aliquots of each *taxon* were powdered using an agata mortar and combined to create one composite sample for each different wood *taxon*.

Two aliquots of each archaeological sample of *Olea europaea*, *Quercus* spp. (evergreen) and *Pinus pinaster* were detached for FT-MIR analysis. As for modern samples, the two aliquots of each archaeological *taxon* were powdered and then combined to create one composite sample to be analysed.

FT-MIR spectra were obtained using an Alpha -R^™^ Spectrometer (Bruker Optics^®^, Germany), with Attenuated Total Reflectance (ATR) module (Platinum-ATR-sampling module, Bruker, Germany), at a wavelength range of 4000–400 cm^-1^ and a resolution of 2 cm^-1^. To improve the signal to noise ratio, 60 spectra were co-added and averaged for each recorded spectrum. Spectral data were background corrected to a reference spectrum obtained prior to every measurement, and some spurious absorptions, such as peaks from atmospheric CO2, could be removed.

#### 3.4.3. Statistical analysis

Partial Least Squares (PLS) regression models were generated using the ParLeS software [90]. This method has been used to acquire predictive cremation temperature models from the FT-MIR spectral intensities in the range 1800–400 cm^-1^ (independent variables, 250 data points). The spectral pre-processing treatments consisted of a light scatter and baseline correction by Standard Noise Variate (SNV), de-noising through a median filter, and mean centering [58]. This pre-processing has been widely used [44, 58, 91, 92] to obtain forecasting models of different environmental and physical variables, using different analytical techniques (FT-IR, Py-CSIA, Py-GC/MS and FTICR/MS) on different natural matrices (e.g., soil, sediment, foodstuff). The protocol of this pre-processing is well exposed in [58]. The Root Mean Squared Error (RMSE) and the Akaike’s Information Criterion (AIC) were used to prevent overfitting and determine the best number of factors (latent variables) for each model. Lastly, the diagnostic spectral regions of the FT-MIR spectra were studied by the combination plot of the Variable Importance for Projection (VIP) values in the spectral range under study. The VIP traces can be useful to identify the independent variables (spectral peaks), that may be linked to the temperature of the cremation. Spurious forecast models due to overfitting were discarded after comparing PLS models calculated with the randomised combustion temperature (cross-validation). The cross-validation model was previously used by [44, 58].

## 4. Results

### 4.1. Anthracological analysis

**Fig 4:**
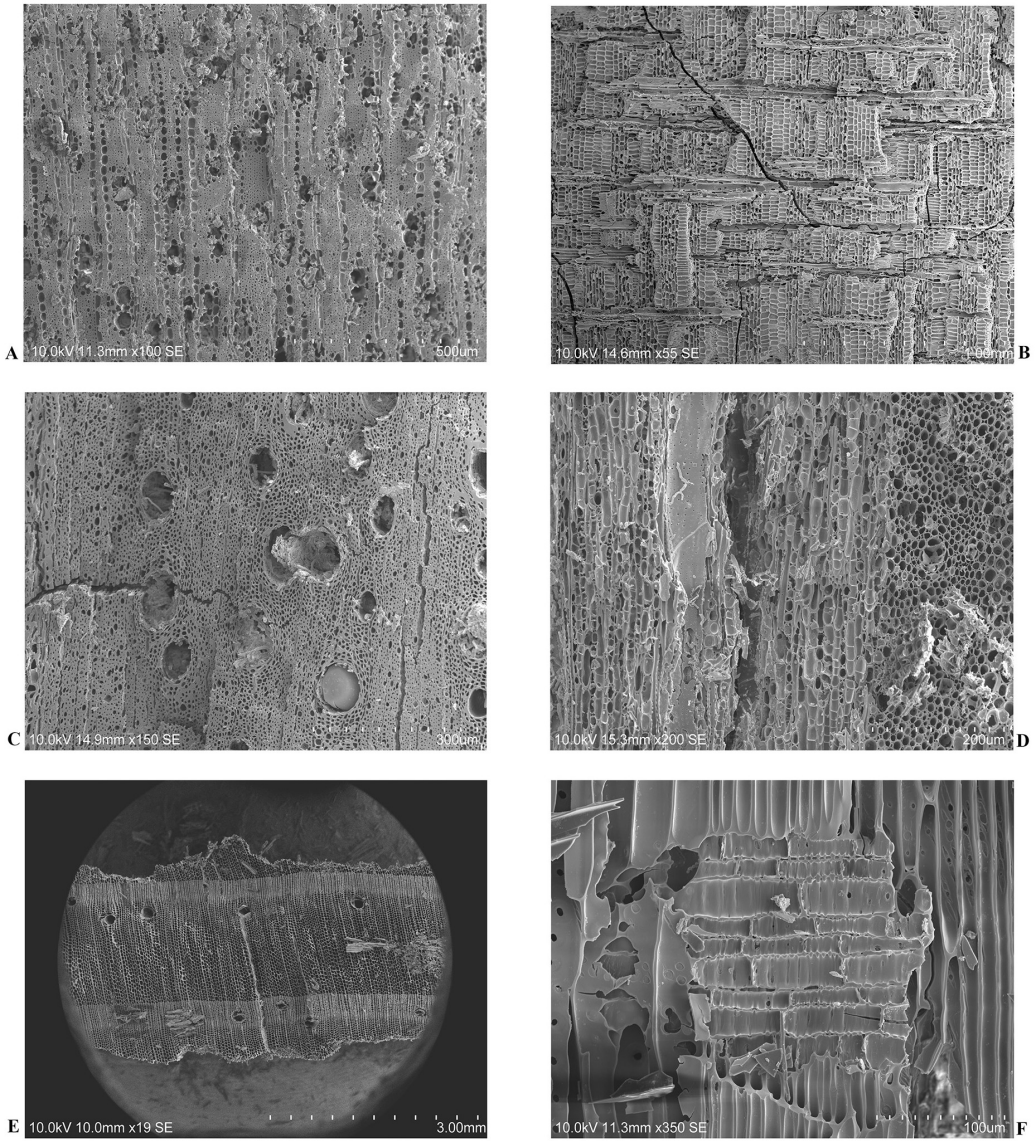
*O*. *europaea*, *Quercus* spp. (evergreen) and *P*. *pinaster* archaeological charcoal samples observed and photographed with SEM. (A, B) Transversal and radial sections of an *O*. *europaea* sample, (C, D) Transversal and tangential sections of a *Quercus* spp. (evergreen) sample, (E, F) Transversal and radial sections of a *P*. *pinaster* sample.

**Fig 5:**
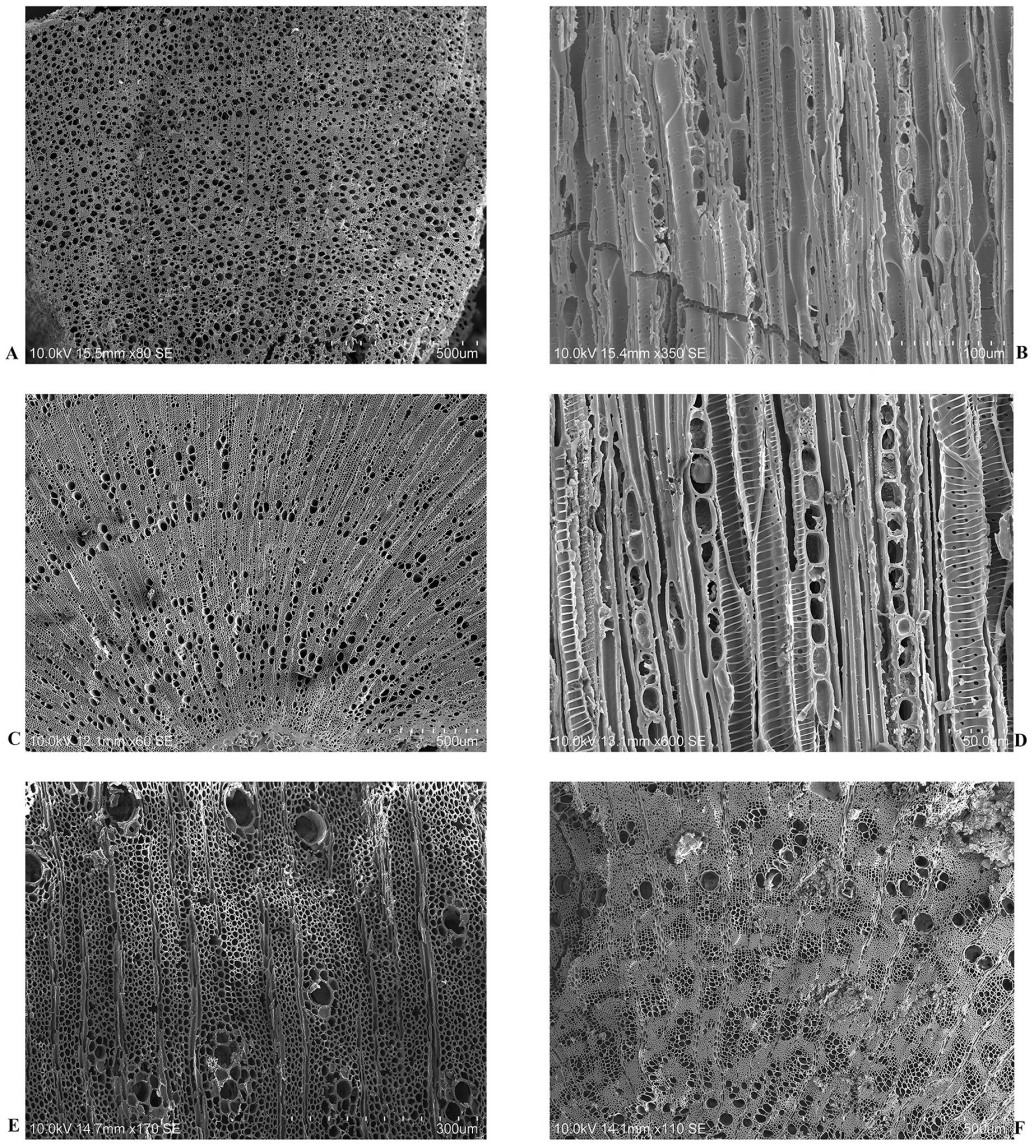
*Cistus* sp., *A*. *unedo*, *F*. cf. *angustifolia* and Fabaceae archaeological charcoal samples observed and photographed with SEM. (A, B) Transversal and tangential sections of a *Cistus* sp. sample, (C, D) Transversal and tangential sections of an *A*. *unedo* sample, (E) Transversal section of a *F*. cf. *angustifolia* sample, (F) Transversal section of a Fabaceae sample.

After the analysis of 754 charcoal fragments from Pit 16, 7 *taxa* were determined at the species level, *Olea europaea*, *Pinus pinaster*, *Fraxinus* cf. *angustifolia*, *Arbutus unedo*, at the section level, *Quercus* spp. (evergreen), at the genus level, *Cistus* sp., and at the family level, Fabaceae. Few charcoal fragments were identified only at the division level, Angiosperm (Table 1, Figs 4 and 5).

**Table 1:** Absolute number (N), relative frequency (%) and relative frequency corrected for ubiquity (%U) of taxa found in Pit 16.

	SU		72		74		TOTAL		
	*TAXA*		N	%	N	%	N	%	%U
	Botanical name	Common name							
***Trees***	*Olea europaea*	Olive	33	15.9	215	39.4	248	**32.9**	**27.6**
	*Quercus* spp. (evergreen)	Evergreen oak	66	317	162	29.7	228	**30.2**	**30.7**
	*Pinus pinaster*	Maritime pine	99	47.6	57	10.4	156	**20.7**	**29**
	*Fraxinus* cf. *angustifolia*	Narrow-leafed ash	0	0.0	10	1.8	10	**1.3**	**0.9**
***Shrubs***	*Cistus* sp.	Rock rose	8	3.8	33	6	41	**5.4**	**4.9**
	Fabaceae	Legume family	2	1.0	14	2.6	16	**2.1**	**1.8**
	*Arbutus unedo*	Strawberry tree	0	0.0	7	1.3	7	**0.9**	**0.7**
	Angiosperm	Undeterminated angiosperm	0	0.0	48	8.8	48	**6.4**	**4.4**
	**Total**		208	100	546	100	754	**100**	**100**
	**Minimum *taxa* number**		**5**		**7**				

**Fig 6:**
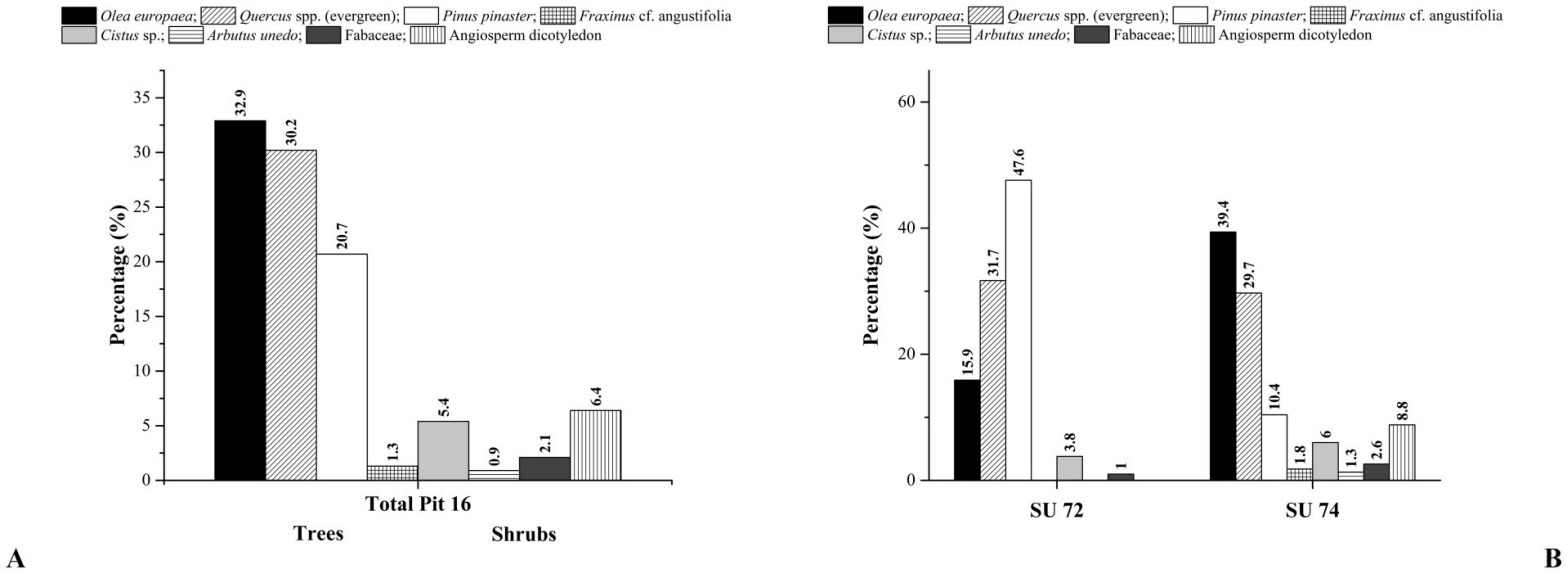
A) Relative frequency of *taxa* found in Pit 16, B) Relative frequency of taxa found in the SU72 and in the SU74.

Three *taxa* are dominant in the anthracological record of Pit 16, featuring the 83.8% and the 87,3%U of total of identified samples: olive tree (*O*. *europaea*), evergreen oak tree (*Quercus* spp. (evergreen)), and maritime pine tree (*P*. *pinaster*) (Fig 4). The most abundant *taxa* are olive (32.9%, 27.6%U) and evergreen oak (30.2%, 30.7%U). Finally, the maritime pine is the third *taxon* in abundance, accounting the 20.7% and the 29%U of the totality (Table 1, Fig 6A). From the SU72, 208 charcoal fragments were recovered, and 5 different *taxa* were identified, while from the SU74, a total of 546 charcoal fragments were recovered and 7 different *taxa* were identified. Moreover, the amount of the 3 main *taxa* is not similar between the two stratigraphic units, with the maritime pine tree being the main *taxon* of the SU72, while in SU74, olive tree is predominant (Table 1, Fig 6B).

These differences become evident by the application of the “ubiquity correction” method, which accounts not only for the absolute number of charcoal fragments, but also for the *taxa* distribution within the samples (Table 1).

Regarding charcoal fragments of *Quercus* spp. (evergreen), samples from both SUs 74 and 72 showed the presence of vitrification. This feature was observed in 85 (37.2%) fragments of this taxon. For the *P*. *pinaster* samples from SU 72, fractures in radial directions (radial cracks) were recorded for a total of 90 (90%) charcoal fragments. Moreover, for 27 charcoal fragments of this species, radial groves in the inner part of tracheids were recorded.

**Fig 7:**
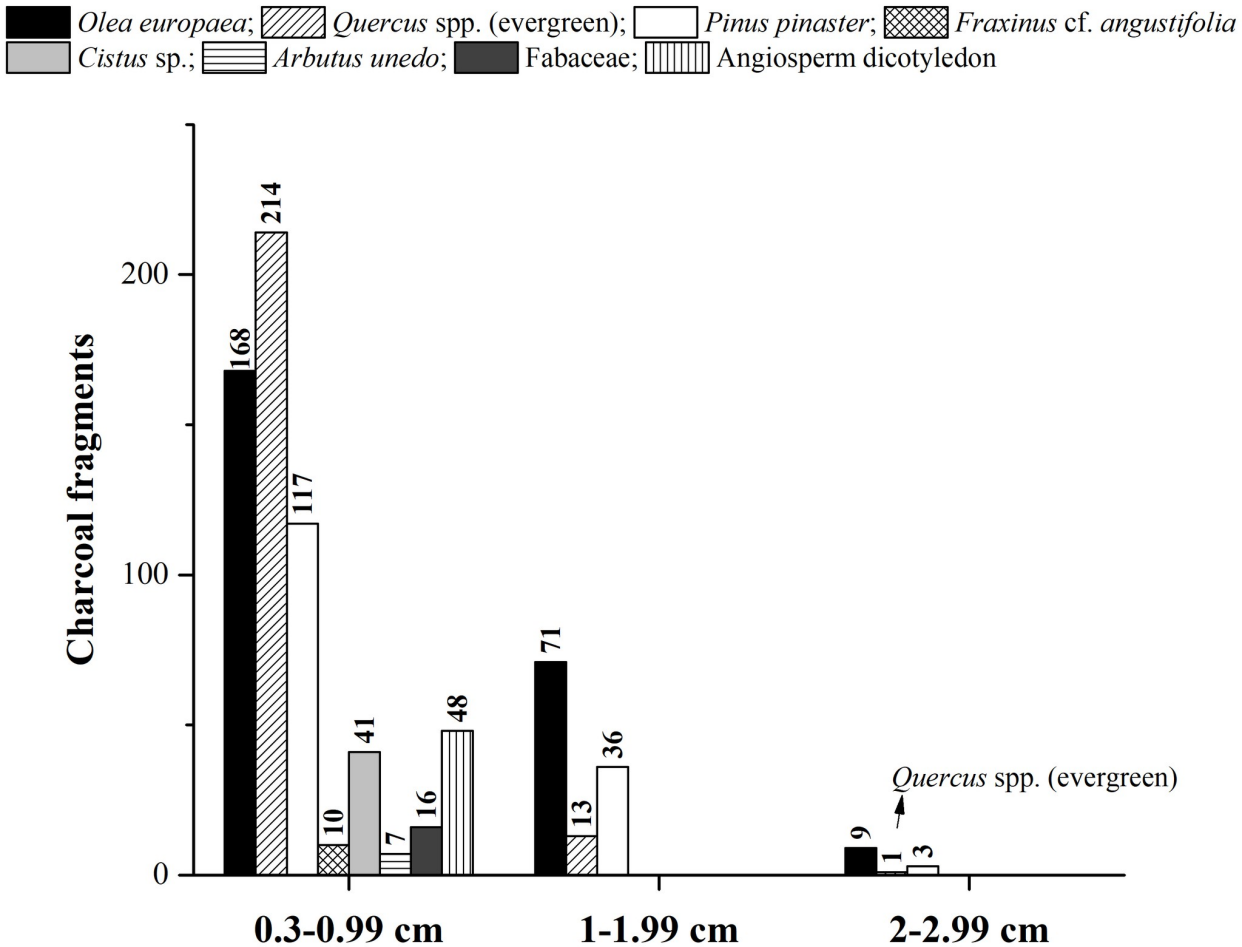
Charcoal size classes identified in Pit 16.

With reference to charcoal size, the olive tree is the species with the highest quantity of the largest charcoal, with 71 fragments with values between 1 and 1.99 cm and 9 fragments between 2 and 2.99 cm., followed by the maritime pine and by the evergreen oak, for which the largest charcoals collected are represented by only 14 fragments (Fig 7).

The remainder of arboreal *taxa* it is represented by few fragments of narrow-leafed ash wood (*F*. cf. *angustifolia*). This *taxon* was recorded only in the SU74, and its presence does not exceed the 1.3% and the 0,9%U (Table 1, Fig 5E). Shrubs were also identified in the anthracological record of Pit 16: rock rose wood (*Cistus* sp.) legume wood (Fabaceae) and strawberry tree wood (*A*. *unedo*), accounting for the 8.4% and the 7,4%U of the total (Fig 5A–5D and 5F). The most present *taxon* is the rock rose (5.4%, 4,9%U), identified on the genus level as *Cistus* sp. (Table 1, Fig 5A–5D). Between shrubs, two fragments of *A*. *unedo* and three of Fabaceae have been identified as twigs, having retained their shape. The values of charcoal size showed that all charcoal fragments belonging to these species were basically smaller, with values between 0.3 and 0.99 cm if compared with the three main *taxa* (Fig 7). Finally, a fava bean seed (*Vicia faba*) was identified in SU74. It was not possible to identify the Angiosperm charcoal fragments beyond the division level due to their state of conservation.

### 4.2. Estimation of the absolute burn temperature

#### 4.2.1. FT-MIR spectroscopy of archaeological samples

**Fig 8:**
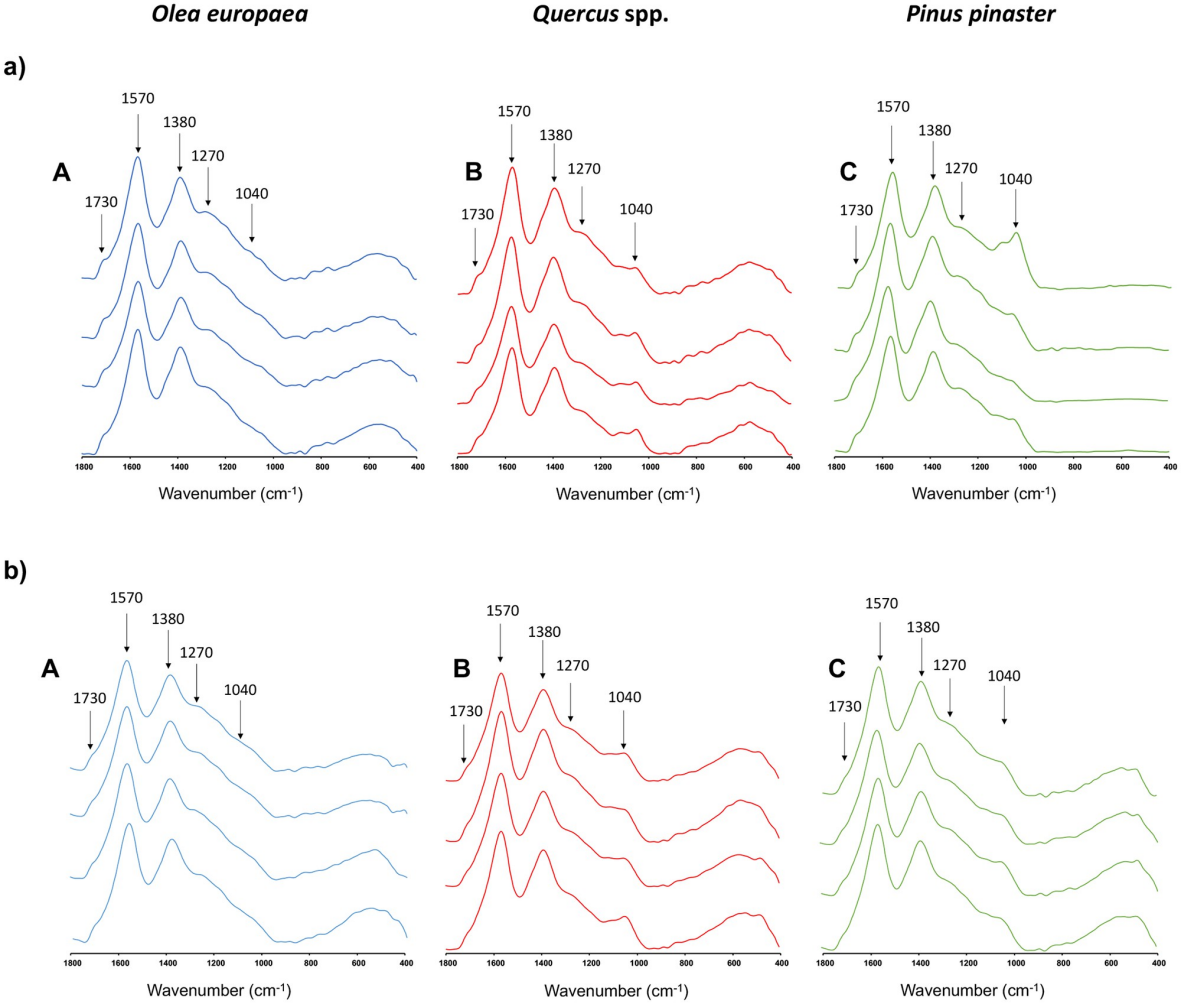
This figure represents the overlay of the infrared spectra to show possible differences among replicates. Medium infrared spectrum (MIR) of archaeological charcoal of *O*. *europaea* (A), *Quercus* spp. (evergreen) (B) and *P*. *pinaster* (C) retrieved from a) SU74 and b) SU72.

Fig 8 displays the infrared spectra, in the region of 1800–400 cm^-1^, of the archaeological charcoal samples from SU74 (Fig 8A) and SU72 (Fig 8B) belonging to each of the three main *taxa* identified after anthracological analysis of archaeological charcoals. In all spectra, different bands were observed, namely the 1570 cm^-1^ band related to the amide groups, the 1380 cm^-1^ band linked to C-O stretching of phenolic OH, the 1730 cm^-1^ band related to stretch of groups C = O, and the bands 1270 and 1040 cm^-1^ belonging to lignin. These results were in line with those observed by other authors [93, 94] for burnt organic matter samples. The band at 1570 cm^-1^ does not have an unequivocal assignation [95], nevertheless it may be referred to the C = C stretching in aromatic and quinone structures, that is highly conjugated to carbonyl group [96]. This band is typical of carbonized, fossilized or highly humified organic matter [97]. The band at 1380 cm^-1^ may be related to unspecific aliphatic structures, as well as hydroaromatic compounds.

#### 4.2.2. FT-MIR spectroscopy and PLS analysis of modern charcoals

**Fig 9:**
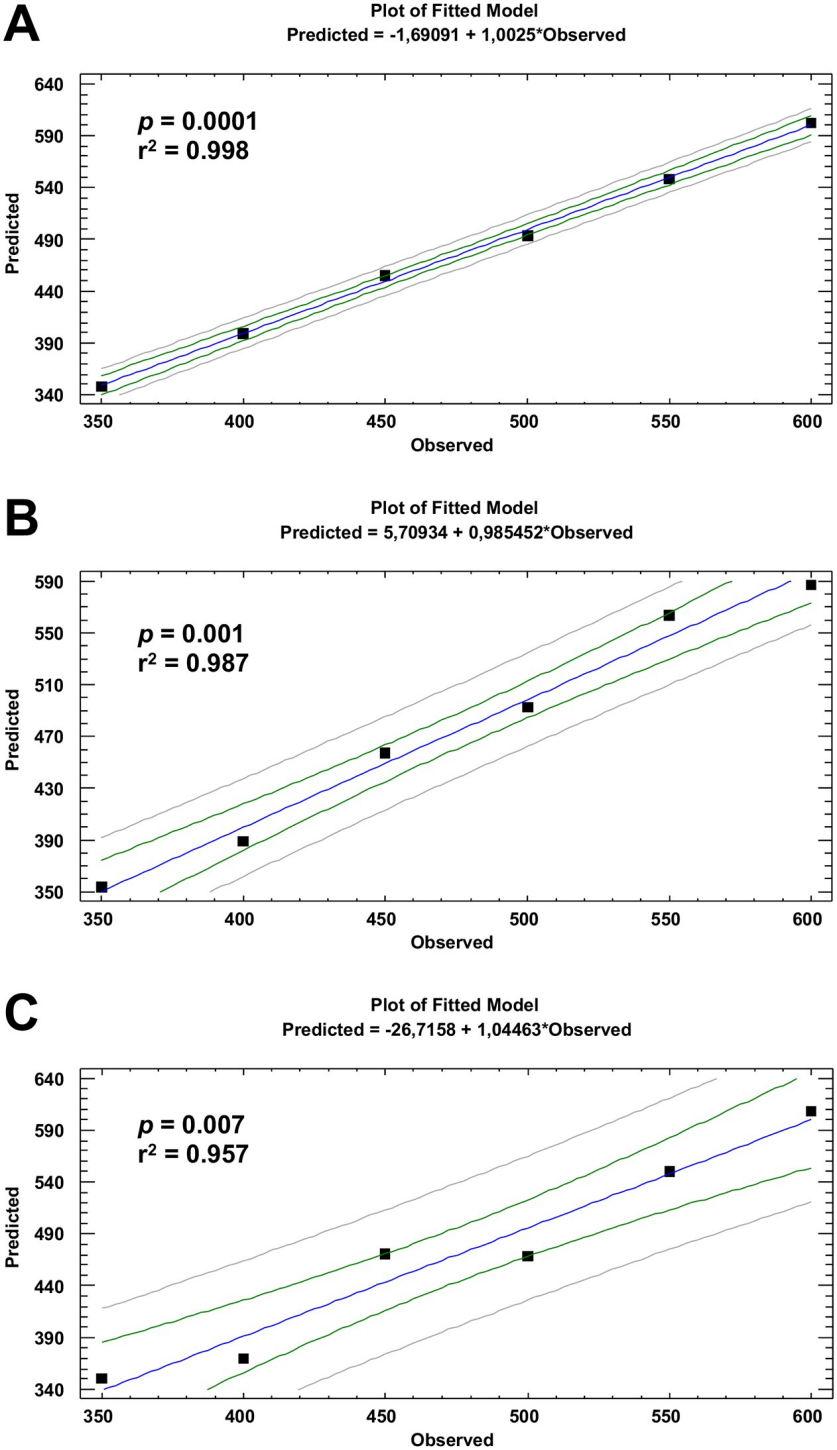
Cross-validation plots for crematory temperature of modern charred woods taxa: A) *O*. *europaea* var. *sylvestris*, B) *Q*. *suber* (evergreen type) and C) *P*. *pinaster*, generated by partial least squares (PLS) regression using infrared spectral as predictors.

**Fig 10:**
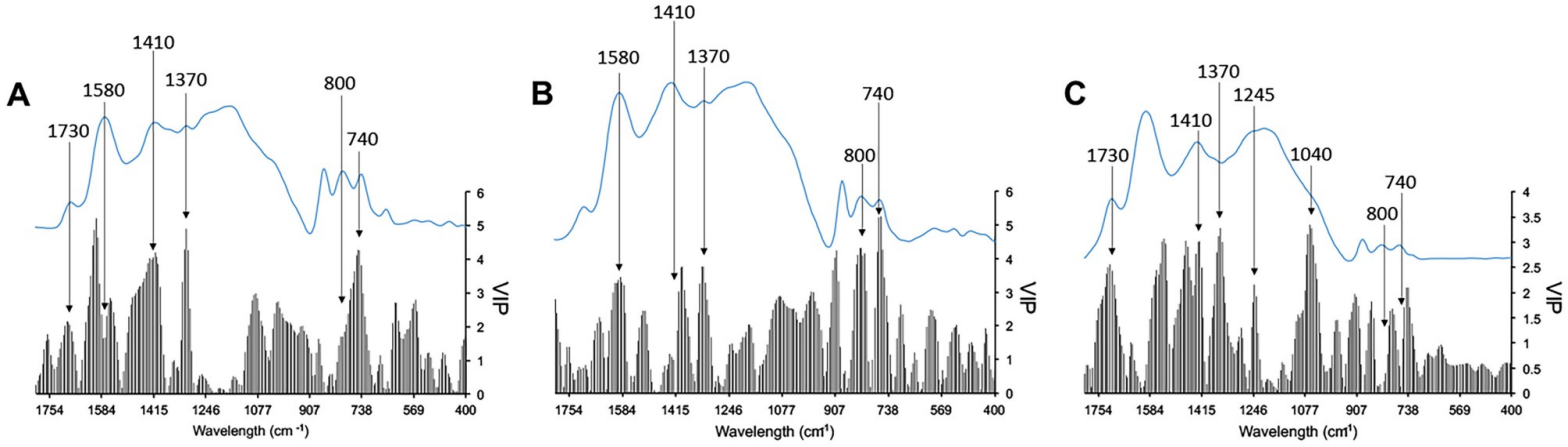
Superposition of variable importance for projection (VIP) of spectral points and average infrared spectra of modern char particles of *O*. *europaea* var. *sylvestris* (A), *Q*. *suber* (B) and *P*. *pinaster* (C).

The cross-validation plots (predicted vs observed values) for the burning temperature of each set of modern wood charred *taxa* are displayed in Fig 9. The PLS models, using exclusively the information contained in a discrete infrared spectra range (1800–400 cm^-1^, 250 data points), successfully predicted the crematory temperature of each *taxon*. Comparison with an alternative model derived from the fully randomised dependent variable that had no connection (*p* > 0.05) with infrared data validated the model’s accuracy. The PLS model of *Olea* showed the highest significant prediction (r^2^ = 0.998, *p* = 0.0000), following by *Quercus* and *Pinus* (r^2^ = 0.987, *p* = 0.001 and r^2^ = 0.957, *p* = 0.007, respectively). In all cases, the RMSE and AIC values suggested the use of four factors for all *taxa*. Fig 10 shows the VIP values for the different *taxa* used to obtain the different PLS models. The superposition of the average infrared FT-MIR spectrum of each *taxon* with the VIP trace indicates the best diagnostic bands to predict the combustion temperature of each *taxon*. The most significant FT-MIR bands to predict the burning temperature in the case of *Olea* model were those positioned at 1730, 1580, 1410, 800 and 740 cm^-1^, while for the *Quercus* model, the predominant bands were located at 1580, 1410, 1370, 800 and 740 cm^-1^. In the case of *Pinus* model, the main bands were identified at 1730, 1410, 1370, 1245 and 1040 cm^-1^.

Each forecasting model was made up of characteristic infrared bands [44]. In the case of the *Olea* samples, the statistic model used the bands at 1730 and 1580 cm^-1^ corresponding to vibrating C = C and C = O bonds, respectively, in the aromatic rings [96]. In addition, the bands at 1410 cm^-1^, related to C–C stretch (aromatic ring) of lignin compounds [98] and 1370 cm^-1^, linked to methyl C–H rock in alkene compounds [99] were also used by the forecasting model of *Olea*. This *taxon* also used the bands at 800 and 740 cm^-1^ which are typical of polysaccharide compounds [100]. The *Quercus* PLS model used the same FT-MIR bands as the model of *Olea*; however, in this case, the band at 1730 cm^-1^ was not determinant. On the other hand, the model of *Pinus* employed the band at 1245 cm^-1^ linked to acidic compounds of the resin [101]. This attribution is further supported by two characteristics bands at 800 and 740 cm^-1^, which indicate the presence of resinous compounds [102]. In addition, in this model, the band at 1040 cm^-1^, that is related to the C–O stretch, displayed a noticeable high VIP value.

#### 4.2.3. Predicted absolute burn temperature of archaeological charcoals

The predicted absolute burn temperature for the archaeological charcoal samples identified in SU74 and SU72 are displayed in Table 2, respectively. The validation of the forecast model by cross-validation process supported its use to determine the burn temperature experimented by archaeological charcoal samples. The temperature value is obtained from the models developed using charred modern wood *taxa*. In SU74, the *Pinus pinaster* showed the highest combustion temperature (576 ± 24°C) in comparison to *Olea europaea* (544 ± 20°C) and *Quercus* spp. (evergreen) (509 ± 6°C). The one-way ANOVA analysis showed that temperatures were statistically significant (95% confidence interval) among different wood *taxa* (Table 2). However, in SU72 (Table 2), the significant (*p*< 0.05) highest crematory temperature is registered by *O*. *europaea* (506 ± 12°C), while *Quercus* spp. (evergreen) and *P*. *pinaster* (492 ± 3°C and 487 ± 3°C, respectively) showed no significant (*p*>0.05) differences among them.

**Table 2:** Mean, minimum and maximum temperature (°C) values of the three main taxa from SU74 and SU72. One-way ANOVA, the different letters indicate significant (*p* < 0.05) mean temperature (°C) differences between taxa according to the Tukey test.

	SU74			SU72		
	Temperature (°C)					
*Taxa*	Mean	Min	Max	Mean	Min	Max
***Olea***	544^b^	520	571	506^a^	494	523
***Quercus***	509^a^	500	516	492^b^	489	494
***Pinus***	577^c^	549	607	487^b^	484	490

## 5. Discussion

### 5.1. Different wood *taxa*

All taxa identified are characteristic of the Mediterranean pine forest and sub-Mediterranean sclerophyllous vegetation [103].

It was possible to identify three main *taxa* in the anthracological record of Pit 16: *O*. *europaea*, *Quercus* spp. (evergreen) and *P*. *pinaster* (Fig 3), with olive and evergreen oak woods prevailing (Table 1, Fig 5A). The presence of the olive wood in the studied context is remarkable, as there are only a few cases where its usage in archaeological human cremations have been reported [33, 37]. Nevertheless, macroremains of this *taxa* were already identified in other types of funerary archaeological contexts from the Middle Neolithic to Bronze period in Iberia [104–107].

On the contrary, use of oak and pine woods is ubiquitous in different human cremations and burials of both prehistoric [24, 27, 29, 30] and historic sites [34, 35, 37, 38, 108, 109]. In all these studies the combustion properties of these two types of wood are mentioned. The simultaneous presence of oak and pine woods in a single funerary site has been observed since prehistory, suggesting a specific human choice [24]. Regarding the identification of *F*. cf. *angustifolia*, it must be emphasized that this species is numerically limited to a small number of charcoal fragments, and it is only present in the SU74 (Table 1, Fig 6B). The presence of ash wood within the Pit, despite being in small amount, is an interesting datum, because it is the only arboreal *taxon* of the anthracological record in addition to the three main *taxa O*. *europaea*, *Quercus* spp. (evergreen) and *P*. *pinaster*. There are plenty of recorded instances of use of ash wood for human archaeological cremations, although its use seems to become consistent rather than sporadic only during the historical time [34, 35, 37, 38, 108]. Regarding the presence of the evergreen *taxa* of *Cistus* sp., *A*. *unedo* and Fabaceae, numerous studies have already widely attested the use of shrubs and small trees in human cremation rituals. The *Cistus* sp. is a small perennial shrub that grows in dry and rocky soils of the Mediterranean area and its presence has been observed in other archaeological human cremations, especially during the Roman period [37, 108]. There are also records about the use of Fabaceae woods and of *A*. *unedo* tree wood in human cremations [38, 108]. *A*. *unedo*, whose presence Pit 16 at Perdigões is limited only to SU 74 (Table 1, Fig 6B), has also been used as a fuel in the Portuguese chalcolithic funerary site of Cabeço da Amoreira (Muge, Portugal) [24].

#### 5.1.1. Different parts from trees

Some anatomical alterations of charcoal from Pit 16, together with charcoal size classes were recorded. The most significant observations have been reported, although they should not be considered as conclusive, since they are not supported by trigonometric and statistical analysis.

Regarding charcoal size classes, results showed that larger charcoals belong to the three main arboreal *taxa* identified (Fig 7). This result seems predictable, considering that the rest of the anthracological record is mainly formed by shrubs, usually with thinner branches than a tree. Nevertheless, we cannot rule out the taphonomic processes undergone by charcoals, and the different degree of fragmentation proper to each species [110–112].

For the charcoal fragments corresponding to the tree *taxa* identified at the anthracological record of Pit 16, i.e. *O*. *europaea*, *Quercus* spp. (evergreen), *P*. *pinaster* and *F*. cf. *angustifolia* (Fig 6A), a strong curvature of the ring was never. This data suggests that large branches or parts of the trunk, together with other minor parts of these trees were possibly employed for the cremation/s. In contrast, the observation of a strong ring curvature for all charcoal fragments belonging to *A*. *unedo* and Fabaceae seem to indicate the use of twigs or small branches of these taxa for the ritual/s. The same observation is attributable to all charcoal fragments of *Cistus* sp., for which a strong ring curvature was also observed.

Overall, the data suggest the possible use of different parts of the tree (larger and small branches, twigs, parts of the trunk), for the made up of the pyre/s formed by charcoal of the pit.

The radial cracks observed in some charcoals of *P*. *pinaster* could be the result of a conspicuous loss of humidity on burned fresh wood [113]; although some authors have argued that these features are not always directly related to green wood [66]. The absence of other signs of degradation, caused by fungi and/or insects [114] on charcoals under study, could be due to the use of *P*. *pinaster* wood shortly after the cutting of the tree.

The reaction wood feature observed on some charcoal fragments of *P*. *pinaster*, namely grooves inside tracheids visible on the longitudinal sections of charcoals, suggests a concentric growth in this part of the tree, developed during its life to prevent fall under its own weight [69].

### 5.2. Woods properties and their management

The characteristics and properties of the identified woods provide information regarding the selection, intentional or not, and use of wood by the Chalcolithic community which prepared the pyre/s deposited in Pit 16.

Let us first discuss the three main *taxa*. Nowadays the burning properties of olive wood are well appreciated, in particular due to its long-lasting fire and the pleasant aroma which derives from its burning. Furthermore, its sacredness is emphasized in the ethnographic literature [109]. It should also be emphasized that, as it is one of the main *taxa* of the anthracological record, this wood might have been intentionally selected by the community who made the ritual, and the selection could have been made for cultural and/or social reasons. As for the oak wood, the literature underlines how it also provides an excellent fuel, long lasting and with high calorific power [115–117]. On the contrary, pine wood burns very quickly, reaching high temperatures rapidly, being usually used for fire lighting [115, 118]. The use of pine wood within funerary rituals for symbolic reasons cannot be excluded, especially given its high resin content, which would produce a pleasant smell during the incineration [37, 108].

Ash wood produces an excellent fuel, and its property of burning, both when is seasoned than when is fresh, is well known. The shrubs identified in the anthracological record of Pit 16, due to small-calibre woods, would seem particularly useful in lighting fires. Moreover, they emanate a pleasant smell during their combustion, and their use in these type of contexts may represent an anthropic symbolic choice [119]. The presence in funerary contexts of rock rose for symbolic reasons has also been contemplated in the literature [120].

Assemblages of concentrated charcoal, especially those coming from a single context, cannot be considered reliable for the reconstruction of the vegetation surrounding the archaeological site [25, 30, 35]. Despite that, the anthracological record of this type of assemblages reflects at least part of the vegetation of the site, representing a very important source of information. In the case of Pit 16 the identified *taxa*, if appropriately compared with the results of pollen analysis at Perdigões [121, 122] and with the current vegetation of the region, offer insights on the possible supply area of woods. These data provide indirect information on the possible area where the primary ritual could have taken place. Pollen analysis carried out on Late Neolithic layers of Perdigões identified the main three *genera* observed in the anthracological record of Pit 16, *i*.*e*., *Olea*, *Pinus* and *Quercus*. Inside the pollen records, the presence of various species belonging to *Cistus* and the presence of Fabaceae species has also been verified [121]. In the Chalcolithic layers of Perdigões, *Quercus* and Oleaceae, the olive family to which belong both *Olea* and *Fraxinus* genera, were identified [122]. Comparing the anthracological record with current vegetation data, we observe that all species present in Pit 16 are characteristic of the typically Mediterranean native vegetation, which surrounds the Perdigões archaeological complex [80, 123, 124]. According to these data, the origin of identified woods could have either been the surroundings of Perdigões or a related area of the same region. The discussion of the results of this study cannot, however, disregard the comparison with other archaeological data, which highlights how the action area of the communities of Perdigões was, actually greater than previously supposed [59]. These data are corroborated by some studies on the mobility of the communities [125, 126], which suggest that the presence of exogenous individuals in the archaeological site may have been large. Reflecting on these evidences, it is therefore necessary to report that wood species identified in the anthracological record of Pit 16 are typical of most of the Mediterranean basin [80, 127, 128].

Based on this data, it is challenging to formulate a single hypothesis concerning the origin of the wood *taxa* collected in the secondary cremation of Pit 16. At this stage it is not possible to exclude that the primary cremation occurred neither in the vicinity of the archaeological site nor in slightly more distant areas.

### 5.3. One context and two rituals?

Starting from the relative frequencies of each species identified in the anthracological record of Pit 16 and their corresponding mean value, obtained by applying the "ubiquity correction" method, results shed light on the original context of archaeological charcoals and on the ritual associated therewith. Differences in the number of charcoals and in the number of *taxa* within the two stratigraphic units (SU72 and SU74) (Table 1, Fig 6B), seem to suggest that woods were intentionally selected to build the pyre/s during the primary ritual of cremation/s. This consideration would strengthen the archaeological data. Moreover, these data allow to hypothesize that the two different stratigraphic units might be two different and subsequent depositional moments, likely as deriving from two different cremations. This hypothesis seems even more reasonable by looking at the differences in the relative frequencies and in the mean values of each identified *taxon* (Table 1, Fig 6A and 6B). Most significant differences in values are related to the species of *O*. *europaea* and of *P*. *pinaster* (Table 1, Fig 6). Regarding the rest of the *taxa*, *F*. cf. *angustifolia* and *A*. *unedo* are only present in SU74 (Table 1, Fig 6B).

These values confirm that the distribution of *taxa* through the two stratigraphic units is not homogeneous (Fig 6B).

### 5.4. Forecasted combustion temperature of charcoals from Pit 16

The clear relationship between FT-MIR data on the test samples and the combustion temperature, in the range tested here, has proved the potential of this technique to correctly recreate burnt temperatures for wood charcoal produced during the primary cremation ceremony.

All archaeological charcoals retrieved in SU74 showed a relative high burn temperature (> 500°C). This result was in line with data reported from other authors [53] who determined the absolute combustion temperature of archaeological charcoal samples from human cremations using reflectance. Nevertheless, in SU72 only the *Olea* charcoal sample particles reached the temperature of 500°C.

The burn temperature calculated by the combination of FT-MIR and chemometrics analysis, in this experiment, may suggest the existence of a single cremation for SU 74 (i.e., the human cremation was made using a mixture of different types of woods in a single pyre). On the other hand, the statistical analysis of the combustion temperature of charcoal sample particles found in SU74 determined the existence of a significant (*p*<0.05) difference among the temperature reached by the different types of wood. This difference may be due to different factors, such as: i) the chemical composition of each type of wood (inflammable compounds like resins or epicuticular waxes); ii) the wood moisture content [129] and/or iii) the resistance to burning (protective cover like suberin) [130]. A single deposition coming from a single pyre, employed for the primary cremation regarding the SU74 could be explained by the significant different combustion temperature of each *taxon*.

In the case of SU72, there were not significant differences between the burning temperature of *Quercus* and *Pinus* archaeological charcoal samples. The highest temperature registered by *Olea* may be due to the low wood moisture content in this kind of wood *taxa*. However, the burn temperature of *Olea* samples from SU72 was significantly different from the rest of the main *taxa* (*Quercus* and *Pinus* samples). In the case of *Pinus* samples, a significant temperature difference of 89°C (Table 2) was observed between the two stratigraphic units SUs 72 (486°C) and 74 (575°C). This fact may reflect the use of relatively green *Pinus*’ individuals in SU72, which usually displayed a relative low concentration of resins and waxes compounds (inflammable), burning at lower temperatures. The possible use of some green woods for the cremation would explain the low combustion temperature of *Pinus* samples from SU 72. In contrast, in SU 74, the highest temperature reached by *Pinus* may reflect the use of seasoned individuals with a relatively noticeable amount of resinous material (high degree of flammability). Therefore, in both cases, the wood seasoning of pine samples could be considered an important *actor* to determine the temperature of combustion.

From the combustion temperature differences of the tree main *taxa* of Pit 16, especially regarding charcoals fragments of *P*. *pinaster*, observed between the two stratigraphic units SUs 72 and 74, we can put forward the hypothesis of the existence of 2 different depositional moments, inside the Pit and consequently coming from two different primary rituals (cremations). This hypothesis was already raised with the anthracological analysis and it corroborates the archaeological data regarding the distinction of the two different stratigraphic units.

The absolute relative temperature reached by *Quercus* charcoals in SU72 is comparable to that one reached in SU74. This may be due to the use of *Quercus suber* wood in both crematory events. The existence of a burning protective cover, like suberin, on trunk would have been able to limit and “average” the combustion temperature of the *Quercus* wood in both crematory events. Moreover, *Q*. *suber* tree displays a relatively high abundance of this species in the Alentejo region [88]. Results showed in this study represent a first step, since it is not taken in account the position of the fuel (top of pyre, center of pyre, outside of pyre), as well as the existence of another type of fuel (e.g., animal fat). Some authors [131] demonstrated the significant role of these kind of factors in the combustion temperature. Therefore, the subsequent experiments will take to account the aforementioned variables.

### 5.5. Culturally selection vs availability selection?

The anthracological study of Pit 16 has proved to be particularly relevant for the understanding of human choices of timber for funerary purposes, demonstrating its importance in discerning the complexity of the human cremation process: do the culturally determined wood selection and the wood selection based on the territorial availability represent two different solutions, two coexistent solutions or are they intrinsic solutions?

If we consider the parameter known as the "principle of least effort" [132], we must take into consideration the possibility that this criterion was applied by the community of Perdigões during the collection of woods for cremation purposes. However, as some authors have already written [30], the taxa availability in the surroundings must be considered a priority for this type of communities. Furthermore, the identification of different types of woods can be interpreted as a decision of the community not to choose one or a few species for specific ritual reasons, but rather indicating a collection based on the easy availability of woods. On the other hand, it is necessary to consider that in the anthracological record of the Pit 16 three woods are dominant: the wild olive, the evergreen oak, and the maritime pine. We know that the first species gains a symbolic significance during historical times, while there is also numerous evidence of the use of the second and the third species in other prehistoric cremations. This is the first time that these kinds of evidence are studied in Perdigões and these results will be essential for the interpretation of the funerary practices in the archaeological site during the Chalcolithic. The data collected so far, and its comparison with other case studies [25, 133] from different prehistoric cremations and burials seems to indicate that cultural choices and choices dictated by the environment go hand in hand in all contexts investigated, emphasizing the intrinsic nature of the two variables.

Answering such a wide question by looking for a singular solution, when human choices were dictated by multiple factors in all periods of communal human existence, past and present, is an unproductive effort.

## 6. Conclusion

The charcoal record of Pit 16 is a mixture of different trees species. The most abundant tree *taxa* are *O*. *europaea*, *Quercus* spp. (evergreen) and *P*. *pinaster*. All the species identified in the study are characteristic of both deciduous and evergreen Mediterranean vegetation. The origin of plants selected seems to indicate the occurring of the human cremation/s either on site, in the vicinity or in an area with access to a similar vegetation.

Regarding the investigation of the charcoals burn temperature, this work has demonstrated the validity of the combination of FT-MIR and chemometric analyses to establish the absolute combustion temperature of archaeological charcoals from a human cremation. Considering both the data from chemometric and anthracological analyses, it was possible to speculate the existence of two different depositional moments inside Pit 16.

As for the understanding of human choices of wood species employed for funerary purposes, results from this study seem to indicate that both cultural choices and the ones dictated by the environment might have driven the Chalcolithic community of Pit 16 in the wood selection for the funerary ritual/s.

This study demonstrated the validity of the use of different approaches, an archaeobotanical, a chemical and a statistical approach, to shed light on the role of woods within the ritual of cremation within the funerary practices present in Perdigões during the Chalcolithic.

## Supporting information

S1 Data (XLSX)
